# Emerging current trends and research focus related to pancreatic cancer metabolism: A bibliometric and visualized analysis

**DOI:** 10.3389/fonc.2022.1009700

**Published:** 2022-11-25

**Authors:** Qian Shen, Chuanlong Zhang, Xiaochen Jiang, Junchen Li, Fudong Liu, Xiyuan Zhang, Ge’er En, Bo Pang

**Affiliations:** ^1^ Guang’anmen Hospital, China Academy of Chinese Medical Sciences, Beijing, China; ^2^ Tianjin University of Traditional Chinese Medicine, Tianjin, China

**Keywords:** pancreatic cancer, metabolism, bibliometric analysis, VOSviewer, CiteSpace

## Abstract

**Background:**

As a malignant digestive system tumor, pancreatic cancer has unique metabolic characteristics. In recent years, the study of pancreatic cancer metabolism is in full swing, which provides a new direction for the treatment of pancreatic cancer patients. However, there is no systematic report of pancreatic cancer metabolism. In this paper, bibliometrics and visualization methods were used to analyze the number of publications, countries/regions, authors, institutions, journals, co-cited references, and keywords of pancreatic cancer metabolism articles, to summarize the research trends and predict research hotspots.

**Methods:**

We searched, screened and downloaded articles on pancreatic cancer metabolism through the Web of Science Core Collection (WoSCC). Using CiteSpace, VOSviewer and Bibliometrix Package to analyze publications, countries/regions, authors, institutions, journals, co-cited references, and keywords of pancreatic cancer metabolism to identify research trends and predict research hotspots.

**Results:**

According to the inclusion and exclusion criteria, a total of 5,255 articles were retrieved during the period 1943-2022. The number of publications on pancreatic cancer metabolism is increasing year by year. The United States (n=1602, 30.49%), China (n=1074, 20.44%), and Italy (n=313, 5.96%) are the three countries with the largest number of publications and citations, and there is close cooperation between countries. LI J (n=55) is the most prolific author. FUDAN UNIV (n=348) is the most published institution. CANCERS (n=118), PLOS ONE (n=93), and CANCER RESEARCH (n=80) are the most popular journals in this field. “Nutriment-deficient environment”, “cancer chemoprevention” and “targeting cancer stem cell” are the main areas of focus. “immunotherapy”, “ferroptosis” and “targeted therapy” are hot keywords in recent years. Taking pancreatic cancer metabolism as an entry point to study the role of traditional Chinese medicine (TCM) mainly focuses on curcumin and resveratrol, lack of broader and deeper research on TCM.

**Conclusions:**

The number of publications on pancreatic cancer metabolism has generally increased, and scholars have generally paid more attention to this field. “immunotherapy”, “ferroptosis” and “targeted therapy” are the current research hotspots. The in-depth study of pancreatic cancer metabolism will provide new ideas for the treatment of pancreatic cancer.

## Introduction

Pancreatic cancer is a malignant tumor of the digestive system with an extremely poor prognosis, with a 5-year survival rate of only 8% (1). It is expected to be the second leading cause of death from cancer in the United States over the next two to three decades (2). At present, the treatment methods for pancreatic cancer mainly include surgery, radiotherapy, chemotherapy, immunotherapy, etc., but still cannot reverse the poor prognosis. As early as 100 years ago, Otto Warburg, a German biochemist, proposed that there are metabolic differences between tumors and normal tissues, and proposed the famous “Warburg” effect. Metabolic reprogramming is one of the important characteristics of tumors, and pancreatic cancer is no exception. There are three main pathways for pancreatic cancer to achieve unlimited proliferation, metastasis, and treatment resistance through metabolism: (1) Intracellular metabolic reprogramming (including glucose, lipids, amino acids, nucleotide, mitochondrial oxidative metabolism, and autophagy); (2) Metabolic crosstalk in tumor microenvironment; (3) Obesity, type 2 diabetes and other human metabolic factors (3–5). In addition, many scholars’ studies have shown that the abnormal metabolism of pancreatic cancer is related to its radioresistance, chemotherapy resistance, and immunosuppression, thus leading to its poor therapeutic effect (6–8). Determining the metabolic characteristics of pancreatic cancer can individualize the treatment effect and prognosis of patients, and provide new ideas for clinical treatment of pancreatic cancer.

Bibliometrics refers to the interdisciplinary science that quantitatively analyzes all knowledge carriers using mathematical and statistical methods. It is a comprehensive knowledge system that integrates mathematics, statistics, and philology and focuses on quantification. In today’s big data era, bibliometrics can help scientists and clinicians sort out the research context and hotspots in a certain research field, predict research trends, and greatly improve their research efficiency (9, 10). Up to now, there are many bibliometric articles related to pancreatic cancer have been published, mainly on the tumor microenvironment (11), stellate cells (12), nanoparticles (13), clinical trials (14), pancreatic cancer in the Middle East and North Africa (15), and machine learning for pancreatic cancer (16), pancreatic neuroendocrine tumors (17), etc. But there is no bibliometric study of pancreatic cancer metabolism.

In this study, based on the data of the WoSCC, we use software such as CiteSpace, VOSviewer, and Bibliometrix package to analyze the number of publications, countries, authors, institutions, journals, references, and keywords of metabolic articles on pancreatic cancer, to sort out the direction and hot spots of metabolic research on pancreatic cancer, to predict the direction of further research.

## Methods

### Data source and search strategy

We collected literature data from the WoSCC database through the Science Citation Index Expanded (SCI-EXPANDED) between 1943 and 2022. The search strategy was: TS=(Neoplasm, Pancreatic OR Pancreatic Neoplasm OR Pancreas Neoplasms OR Neoplasm, Pancreas OR Neoplasms, Pancreas OR Pancreas Neoplasm OR Neoplasms, Pancreatic OR Cancer of Pancrea OR Pancreas Cancers OR Pancreas Cancer OR Cancer, Pancreas OR Cancers, Pancreas OR Pancreatic Cancer OR Cancer, Pancreatic OR Cancers, Pancreatic OR Pancreatic Cancers OR Cancer of the Pancreas OR Pancreatic Carcinoma OR Pancreatic Malignancy) AND TS=(Metabolism OR Metabolic OR Metaboly). Only articles and reviews were included in the analysis and the article language was set to English. To avoid bias, the literature search and download were completed by two authors within one day on July 19, 2022. After data deduplication and normalization, the literature including full records and cited references were exported in plain text (**Supplemental File 1**) and tab-delimited formats (**Supplemental File 2**).

### Bibliometric analysis

Microsoft Office Excel 2019 (Microsoft, Redmond, Washington, USA) was used to process the data and construct a polynomial regression model to predict the annual growth trend of publications.

CiteSpace (version 5.8.R3) is software that focuses on and analyzes the potential knowledge contained in scientific analysis. It is a citation visualization analysis software gradually developed under the background of scientometrics and data visualization (18). Citespace software is used to perform cluster analysis of keywords and references, and produce timeline views, dual-map overlay, and the strongest citation bursts. In this study, CiteSpace parameters included were as follows: Time slicing from January 1943 to July 2022, years per slice. The selection criteria for the analysis of references co-citation was set as g-index (k=25).

VOSviewer (version 1.6.18) is a computer program for constructing and viewing bibliometric maps (19). It was used to perform co-occurrence analysis of countries/regions, institutions, authors, and keywords in different ways, including network visualization, overlay visualization, and density visualization.

The Bibliometrix 4.1.0 Package (20) (https://www.bibliometrix.org) is an established tool based on the R language. It is used to analyze publications, countries/regions, authors, and institutions of pancreatic cancer metabolism.

## Results

### Annual growth trend of publications

**Figure 1:**
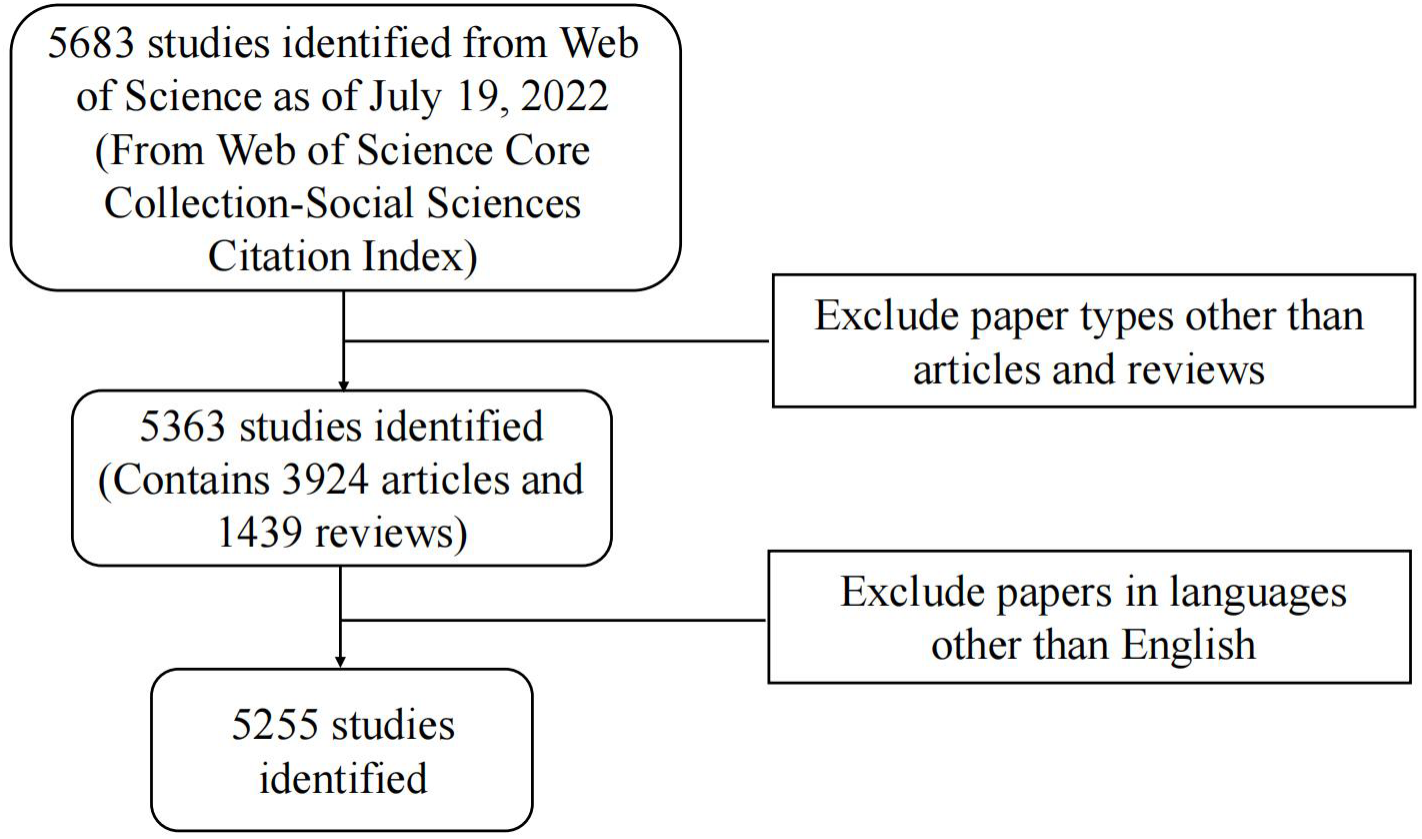
Study flow diagram.

**Figure 2:**
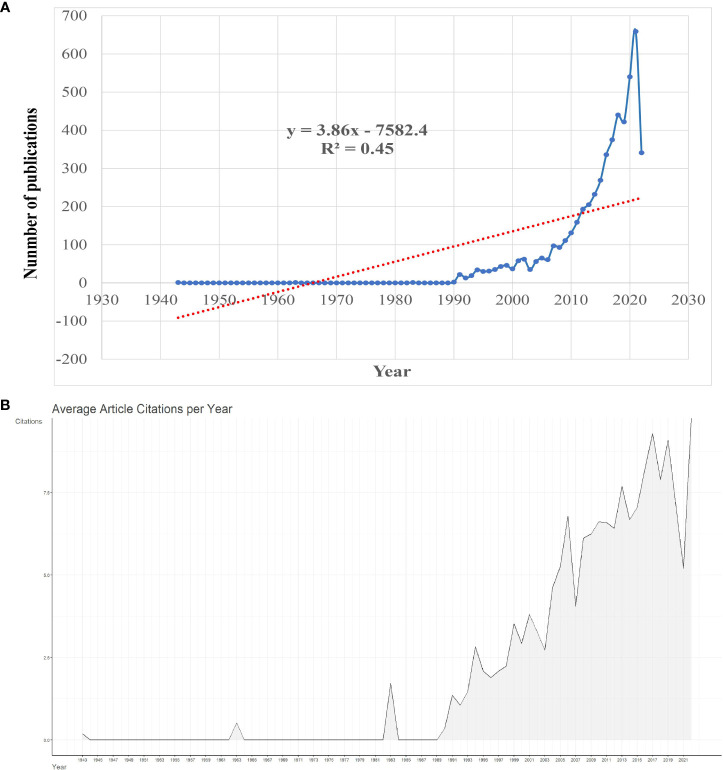
Overview of publications on pancreatic cancer metabolism. **(A)** Global annual output trends; **(B)** Global annual citations trends.

The flow of literature data analysis is shown in **Figure 1**. According to the inclusion and exclusion criteria, a total of 5255 articles in the WOSCC database were included in this study, including 3845 articles and 1410 reviews. As can be seen from the polynomial regression model, since 1943, the study of pancreatic cancer metabolism has increased year by year, with an average annual growth rate of 7.66% (R^2 =^ 0.45). We divide the research on pancreatic cancer metabolism into embryonic stage (1943-1989), rapid growth stage (1990-2008), and mature development stage (2009-2022) according to the trend of annual publication volume and an average number of citations (**Figure 2A**). At the embryonic stage, only one paper was published each in 1943, 1963, and 1983. In the rapid growth stage, the annual number of articles published is between 10 and 100, with an average annual growth rate of 44.16 articles and an average annual growth rate of 22.39%. In the mature development stage, the annual number of publications exceeded 100, with an average annual growth rate of 315.21, with an average annual growth rate of 8.35%, accounting for 83.98% of the total number of publications from 1943 to 2022. The average number of citations per paper was 40.68, and the annual average number of citations showed an overall upward trend (**Figure 2B**).

### Contributions of countries/regions to global publications

**Figure 3:**
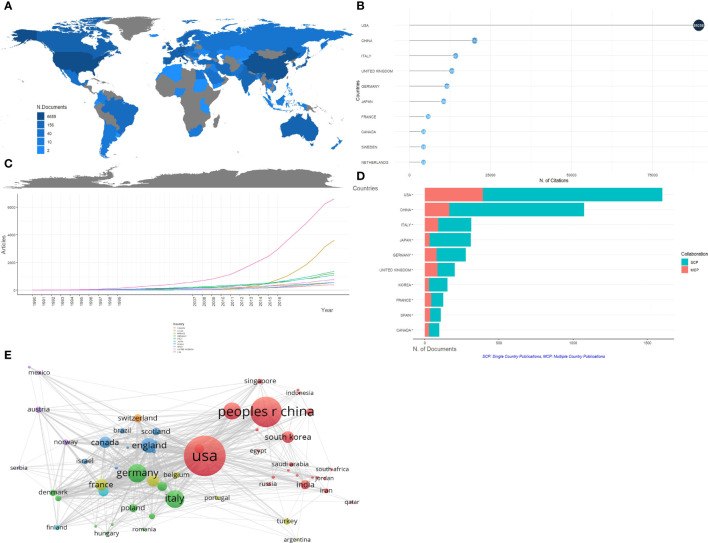
Analysis of countries/regions concerning research on pancreatic cancer metabolism. **(A)** Geographical distribution of global output; **(B)** Visualization map of the top 10 productive countries/regions; **(C)** Annual output trend of the top 10 productive countries/regions; **(D)** Countries/regions in the top 10 of centrality; **(E)** Visual cluster analysis of cooperation among countries/regions.

There are 71 countries engaged in the field of pancreatic cancer metabolism research, among which the top 10 countries with the number of publications are listed in **Table 1**. The distribution of a published quantity of each country is shown in **Figure 3A**. The USA, China, and Italy ranked in the top three, accounting for 30.49%, 20.44%, and 5.96% of the total publications respectively. Moreover, they are also the most cited countries, which indicates that these three countries play an important role in the study of pancreatic cancer metabolism (**Figure 3B**). Looking at the time evolution of the publication volume of each country, it is not difficult to see that the publication volume of each country has increased significantly since 2007 (**Figure 3C**). Single Country Publications (SCP) and Multiple Country Publications (MCP) indicate the number of co-authored papers with authors of the same nationality and different nationalities, and MCP Ratio indicates the rate of international collaboration. As we can see in **Figure 3D**, the United Kingdom, France, and Spain have higher levels of international cooperation. The country visualizations show close cooperation among countries (**Figure 3E**).

**Table 1:** Global Publications in the 10 most productive countries/regions.

Rank	Country	Articles	Centrality	SCP	MCP	Frequency	MCP_Ratio	Average citations
1	USA	1602	0.85	1210	392	0.303	0.245	55.72
2	China	1074	0.04	909	165	0.203	0.154	18.72
3	Italy	313	0.08	222	91	0.059	0.291	45.46
4	Japan	310	0.03	277	33	0.059	0.106	34.15
5	Germany	277	0.08	197	80	0.052	0.289	41.74
6	United Kingdom	202	0.08	117	85	0.038	0.421	64.78
7	Korea	153	0.04	125	28	0.029	0.183	21.90
8	France	124	0.05	81	43	0.023	0.347	46.68
9	Spain	108	0.05	71	37	0.02	0.343	34.13
10	Canada	97	0.05	68	29	0.018	0.299	45.20

### Author contributions

**Figure 4:**
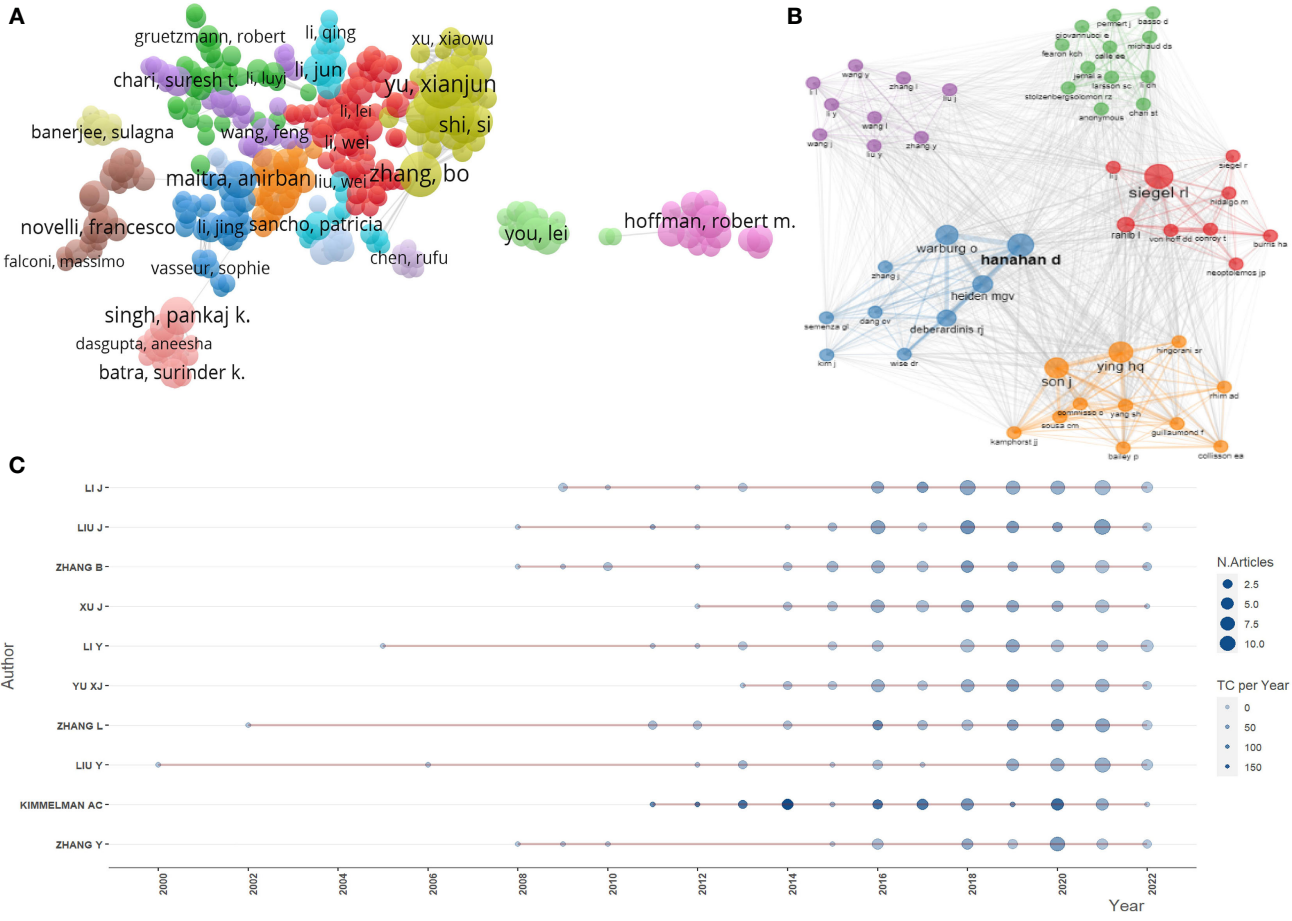
Visualization of active institutes and authors analysis. **(A)** Network map showing cooperation among authors; **(B)** Cluster analysis of cooperation among institutes; **(C)** Timeline distribution of the top 10 most productive authors.

According to our results, a total of 26,648 authors were involved in the study of pancreatic cancer metabolism (**Figure 4A**). The top three authors are LI J, LIU J, and ZHANG B, all of whom are Chinese (**Table 2**). The top 10 authors published 402 papers, accounting for 7.6% of the total published papers. According to the publication time of each author, among the authors with high production, LIU Y published his first paper in 2000. He has been engaged in the research of pancreatic cancer metabolism for more than 20 years. Other authors have focused on the field since 2008 (**Figure 4B**). Authors involved in pancreatic cancer metabolism have collaborated closely with each other, forming four major networks of collaboration (**Figure 4C**).

**Table 2:** Top 10 most productive authors in the field of pancreatic cancer metabolism.

Rank	Author	Articles	Articles fractionalized	Total citation	H-index	Production year start
1	LI J	55	6.01	1267	19	2009
2	LIU J	46	4.68	1941	22	2008
3	ZHANG B	44	4.56	1485	20	2008
4	YU XJ	41	3.96	988	19	2013
5	XU J	40	4.26	1035	19	2012
6	LI Y	39	4.98	537	16	2005
7	ZHANG L	38	5.29	1671	21	2002
8	LIU Y	36	4.67	844	13	2000
9	KIMMELMAN AC	33	5.93	7663	24	2011
10	ZHANG Y	30	3.45	636	12	2008

### Contribution of institutions

**Figure 5:**
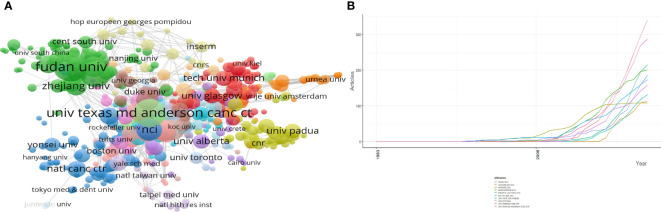
Analysis of institutions involved in pancreatic cancer metabolism. **(A)** Cluster analysis of cooperation among institutes; **(B)** Outputs trend of the top 10 most productive institutes.

There are 4,675 institutions involved in the study of pancreatic cancer metabolism, the top 10 of which are shown in **Table 3**. The top three institutions are FUDAN UNIV, UNIV TEXAS MD ANDERSON CANC CTR and SHANGHAI JIAO TONG UNIV, whose articles account for 16.38% of the total publications (**Figure 5A**). As can be seen from **Figure 5B**, since 2008, the number of papers published by various institutions on pancreatic cancer metabolism has gradually increased, indicating that this field has attracted more and more attention from scholars.

**Table 3:** Top 10 most productive institutions in the field of pancreatic cancer metabolism.

Rank	Institution	Articles	Production year start
1	FUDAN UNIV	348	2008
2	UNIV TEXAS MD ANDERSON CANC CTR	288	2007
3	SHANGHAI JIAO TONG UNIV	225	2008
4	JOHNS HOPKINS UNIV	201	2001
5	UNIV CALIF LOS ANGELES	186	1996
6	UNIV MICHIGAN	175	1996
7	UNIV NEBRASKA MED CTR	158	2007
8	SUN YAT SEN UNIV	132	2008
9	HARVARD MED SCH	115	1998
10	HARVARD UNIV	108	1998

### Core journals

**Figure 6:**
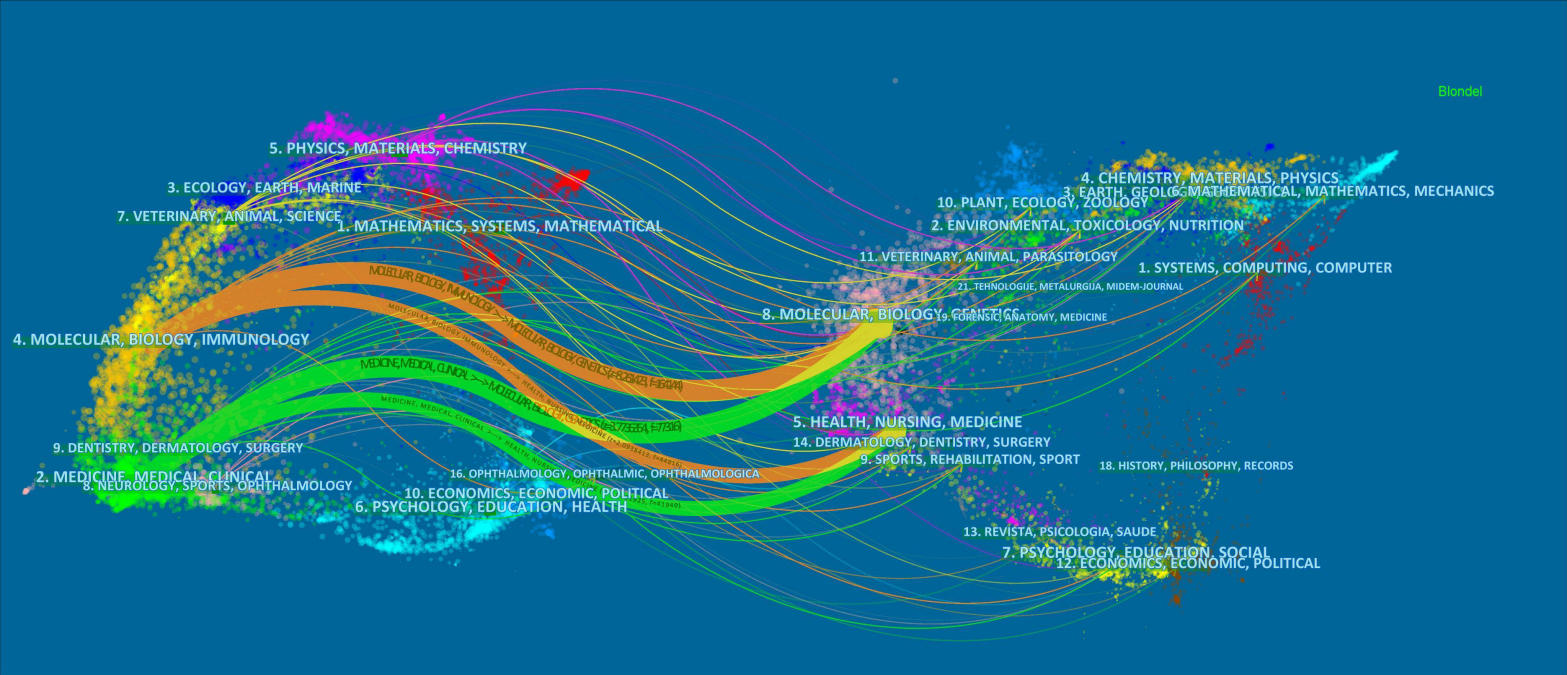
The dual-map overlay of articles cited on pancreatic cancer metabolism. (The left side was the citing journal, the right side was the cited journal, and the line path represents the citation relationship).

Between 1943 and 2022, a total of 1,263 journals published pancreatic cancer metabolism-related articles. The top 10 journals are shown in **Table 4**, where about 14.29% of the articles were published. CANCERS (published 118 articles, cited 1,128 times) ranks first in publication volume, but the most cited journal is CANCER RESEARCH (published 80 articles, cited 4,969 times). The double map superposition shows four main citation paths (**Figure 6**). The published papers are mainly published in journals in Molecular, Bioligy, Immunology, medicine, medical and clinical fields. The cited articles mainly focus on journals in the fields of Molecular, Bioligy, genetics, health, nursing, and medicine.

**Table 4:** The top 10 core journals in the field of pancreatic cancer metabolism.

Rank	Journals	Articles	H-index	Total citation	IF (2021)	Production year start
1	CANCERS	118	16	1128	6.575	2016
2	PLOS ONE	93	29	3070	3.752	2010
3	CANCER RESEARCH	80	42	4969	13.312	1993
4	INTERNATIONAL JOURNAL OF MOLECULAR SCIENCES	78	20	1617	2.866	2012
5	ONCOTARGET	75	32	2618	–	2014
6	PANCREAS	72	27	1796	3.243	1990
7	FRONTIERS IN ONCOLOGY	71	17	906	5.738	2016
8	SCIENTIFIC REPORTS	61	20	1109	4.996	2014
9	CLINICAL CANCER RESEARCH	52	34	3598	13.801	1995
10	CANCER LETTERS	51	25	1891	9.756	1995

### Co-cited references

**Figure 7:**
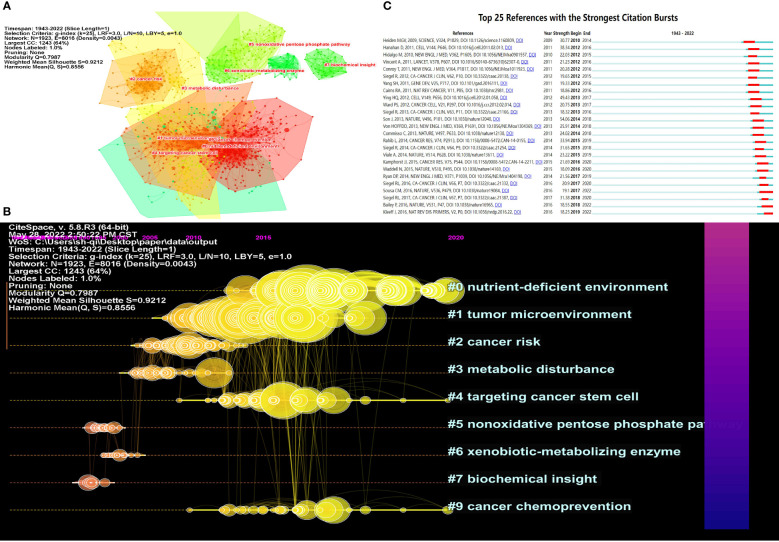
Visualization of co-cited references analysis. **(A)** Cluster analysis of co-cited References; **(B)** The top 10 kinds of literature with the highest number of citations; **(C)** Top 25 references with the strongest citation bursts.

According to our results, a total of 23,6377 references were involved in 5,255 articles on pancreatic cancer metabolism. The top 10 references of pancreatic cancer metabolism articles are shown in **Table 5**. We can see that most of these journals with the highest citation volume are published in top-quality journals such as Science, Nature, and Cell. We used Citespace software to cluster the references and selected the top 10 clusters with the highest K value for visualization. The modularity Q was 0.7987, showing that the network was reasonably divided into the loosely coupled cluster. The mean silhouette value was 0.9212, indicating that the clusters had acceptable homogeneity. The top 10 distinct clusters were marked as “#0 tnutrient-deficient environment”, “#1 tumor microenvironment”, “#2 cancer risk”, “#3 metabolic disturbance”, “#4 targeting cancer stem cell”, “#5 nonoxidative pentose phosphate pathway”, “#6 xenobiotic-metabolizing enzyme”, “#7 biochemical insight”, “#8 fatty pancrea” and “#9 cancer chemoprevention” (**Figure 7A**). We then visualized the clustering results over time, and we found that Through insight, studies through pancreatic cancer metabolism in the early stage include “nonoxidative pentose phosphate pathway”, “xenobiotic-metabolizing enzyme” and “biochemical insight”. At present, research on pancreatic cancer metabolism is concentrated on “nutriment-deficient environment”, “cancer chemoprevention” and “targeting cancer stem cell” (**Figure 7B**). Moreover, the top 25 references with the strongest citation bursts are shown in **Figure 7C**, indicating the evolution of metabolic references in pancreatic cancer.

**Table 5:** The 10 most cited articles in the field of pancreatic cancer metabolism.

Rank	Cited references	Citations	Authors	Journal	Type	Publication year
1	Hallmarks of cancer: the next generation	402	HANAHAN D	CELL	Review	2011
2	Understanding the Warburg effect: the metabolic requirements of cell proliferation	318	HEIDEN MGV	SCIENCE	Review	2009
3	Glutamine supports pancreatic cancer growth through a KRAS-regulated metabolic pathway	316	SON J	NATURE	Experiment	2013
4	Oncogenic Kras maintains pancreatic tumors through regulation of anabolic glucose metabolism	301	YING HQ	CELL	Experiment	2012
5	On the origin of cancer cells	264	WARBURG O	SCIENCE	Experiment	1956
6	Projecting cancer incidence and deaths to 2030: the unexpected burden of thyroid, liver, and pancreas cancers in the United States	263	RAHIB L	CANCER RES	Epidemiological Study	2014
7	Cancer statistics, 2019	199	SIEGEL RL	CA-CANCER J CLIN	Epidemiological Study	2019
8	FOLFIRINOX versus gemcitabine for metastatic pancreatic cancer	186	CONROY T	NEW ENGL J MED	Clinical Trial	2011
9	Increased survival in pancreatic cancer with nab-paclitaxel plus gemcitabine	167	VON HOFF DD	NEW ENGL J MED	Clinical Trial	2013
10	Genomic analyses identify molecular subtypes of pancreatic cancer	162	BAILEY P	NATURE	Experiment	2016

### Analysis of keywords

**Figure 8:**
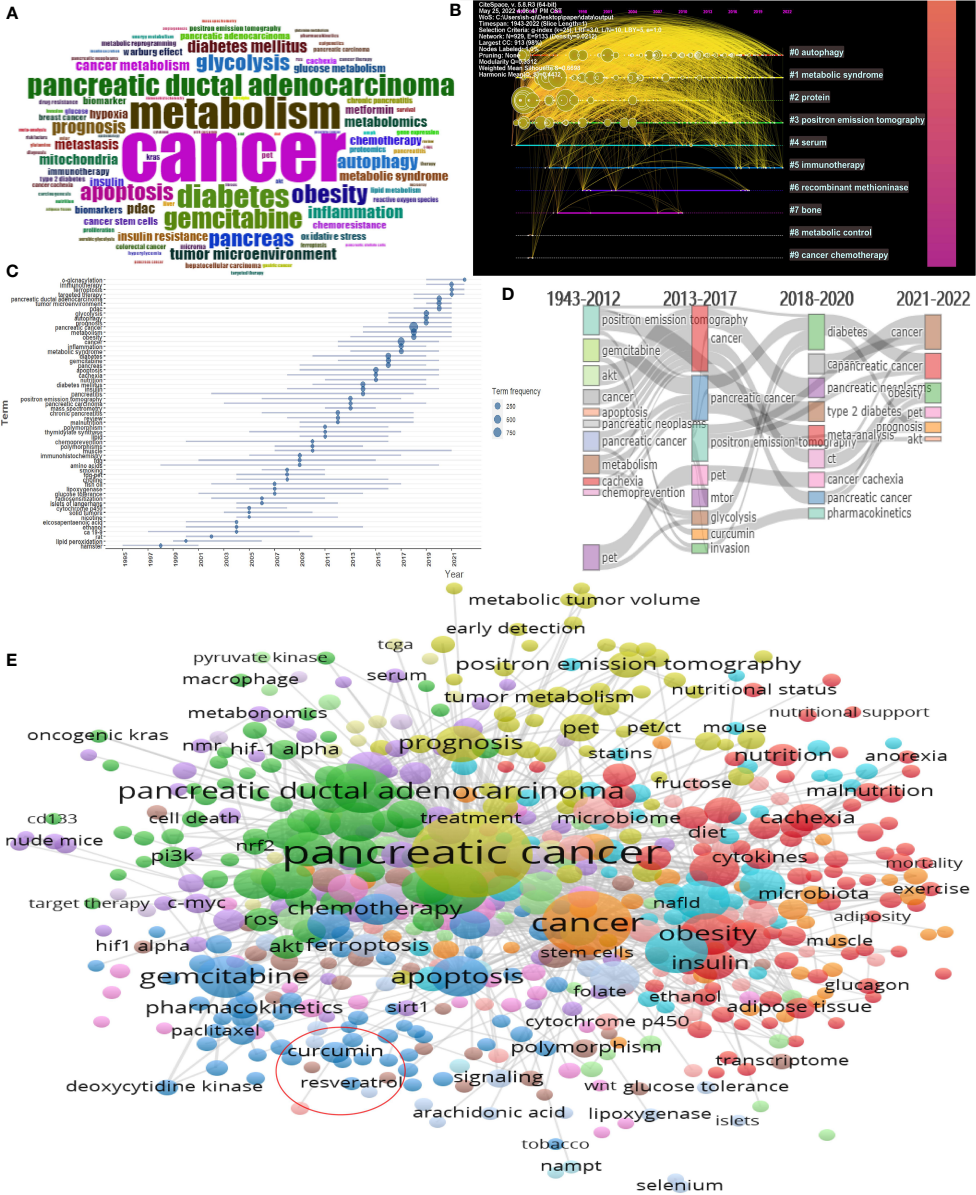
Visualization of keywords analysis. **(A)** Visualization of all keywords; **(B)** Timeline distribution of cluster analysis of keywords; **(C)** Timeline diagram of the first occurrence of a keyword **(D)** Sankey diagram of the keywords evolution; **(E)** Cluster analysis of keywords.

A total of 8738 keywords proposed by the authors in the article were included in this study (**Figure 8A**). We perform cluster analysis on keywords and select the top 10 with the highest K value for visualization (**Figure 8B**). We can see that “autophagy”, “metabolic syndrome”, and “positron emission tomography” are important areas of current pancreatic cancer metabolism research. “immunotherapy” is an emerging research hotspot recently. By studying the time when the keywords first appeared and the time flow process of the keywords, we can see that the keywords gradually changed from “gemcitabine”, “chemoprevention” and “positron emission tomography” to “cachexia”, “diabetes” and “glycolysis” in the early stage. In the past two years, there have been new hot keywords such as “immunotherapy”, “ferroptosis” and “ targeted therapy”(**Figures 8C**, **D**). Through our further research on keywords, we found that there are few related studies on Chinese medicine and pancreatic cancer metabolisms, such as curcumin (occurrences: 22, total link strength: 51) and resveratrol (occurrences: 7, total link strength: 15) (**Figure 8E**).

## Discussion

In this study, we included a total of 5255 articles on pancreatic cancer metabolism in the WOS database according to inclusion and exclusion criteria. Subsequently, we used Citespace, Vosviewer, and other software to perform a bibliometric and visual analysis of articles on pancreatic cancer metabolism.

**Figure 9:**
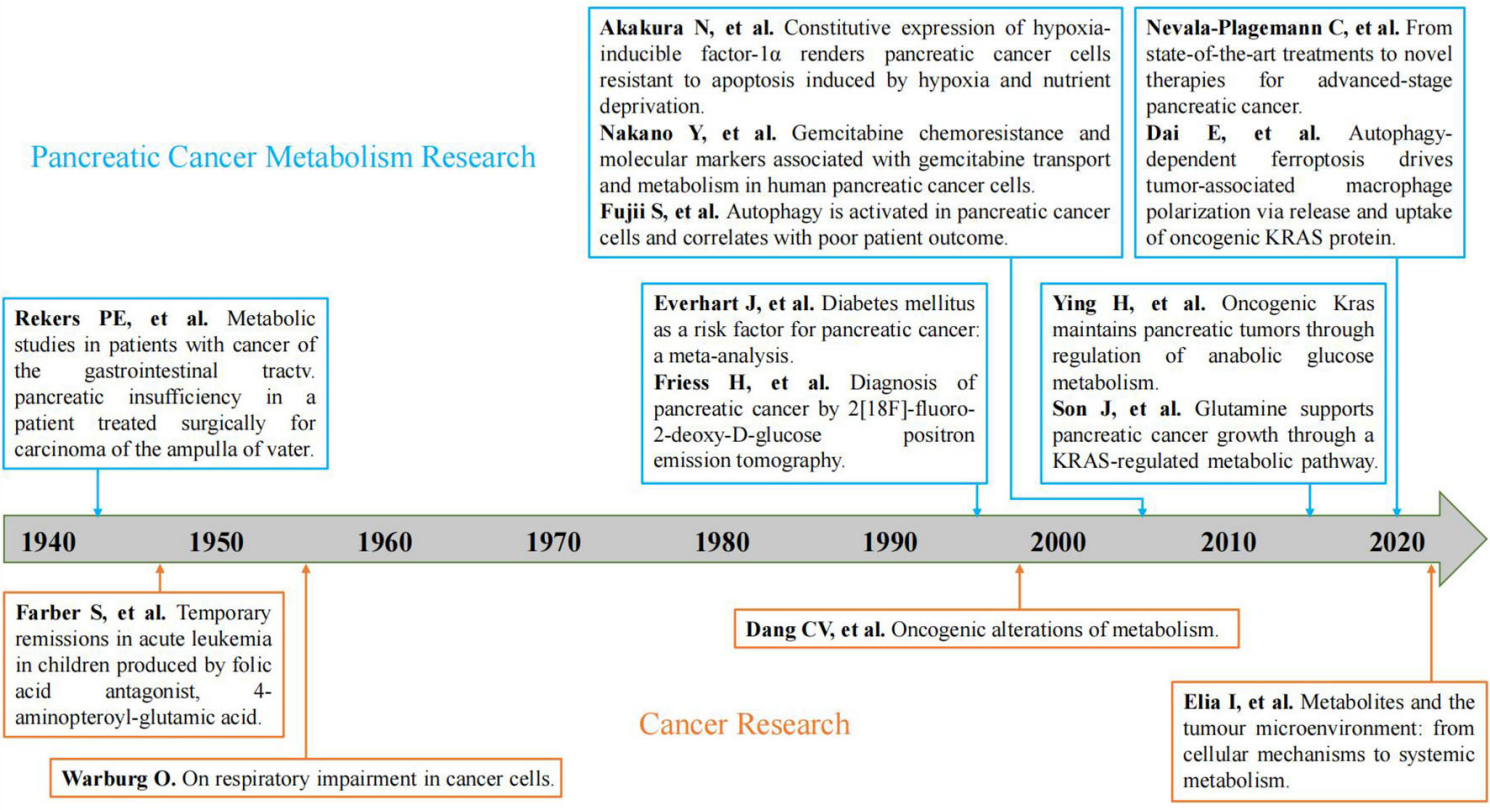
Development of representative studies of tumor metabolism and pancreatic cancer metabolism. (The blue is a representative study of pancreatic cancer metabolism; The orange is a representative study of tumor metabolism (21)).

From our research results, we can see that the annual publication volume and average annual citation frequency of pancreatic cancer metabolism are increasing year by year. The published volume of pancreatic cancer metabolism has gone through the embryonic stage (1943-1989), rapid development stage (1990-2008), and mature development stage (2009-2022) (**Figure 9**). In the embryonic stage, the “Warburg” effect proposed by Otto Warburg (22) and the antifolic acid therapy proposed by Sidney Farber in childhood acute leukemia (23) has laid a foundation for the study of tumor metabolism. In 1943, Rekers et al. (24) first described the use of pancreatic enzymes to relieve metabolic disorders in postoperative patients with pancreatic cancer through a case report, which provided a starting point for the study of pancreatic cancer metabolism. In the 1990s, the link between oncogenes, tumor suppressors and metabolism began to emerge, which triggered scholars’ interest in cancer metabolism and greatly increased the research on pancreatic cancer metabolism (25). During this period, Everhart et al. (26) identified diabetes as a risk factor for the development of pancreatic cancer through meta-analysis. Fujii et al. (27) first explored the relationship between pancreatic cancer and autophagy from the perspective of clinicopathology. Akakura et al. (28) demonstrated that pancreatic cancer cells resist apoptosis by inducing hypoxia and nutritional deprivation through hypoxia-inducible factor 1 alpha (HIF-1 alpha). FRIESS et al. (29) proposed 2[F-18]-fluoro-2-deoxy-D-glucose positron emission tomography to diagnose pancreatic cancer. Nakano et al. (30) found Equilibrative nucleoside Transporter-1 (hENT1) in humans, Deoxycytidine kinase (dCK), ribonucleotide reductase catalytic subunit M1 (RRM1), and RRM2 is associated with gemcitabine resistance acquired in pancreatic cancer cells. For nearly twenty years, places of the tumor microenvironment as a tumor metabolism plays an important role, scholars also gradually from cancer itself metabolism and tumor microenvironment combined with the study of metabolism (31). Moreover, immunotherapy has entered the stage of cancer treatment. Dai et al. (32) found that autophagy-dependent ferric death drives tumor-associated macrophage polarization through the release and uptake of oncogenic KRAS protein in pancreatic cancer. Moreover, Ying et al. (33) and Son et al. (34) took KRAS as the starting point to investigate the details of metabolic reprogramming in pancreatic cancer. Nevala-plagemann et al. (35) summarized the latest treatment strategies for pancreatic cancer based on the results of the current study.

The USA is not only the country with the largest number of single publications but also the country with the most multiple publications, which shows that American scientists are good at communicating and cooperating with various countries. Except for China, the top 10 countries with the number of publications are all developed countries. This indicates that China stands at the forefront of the developing countries in the field of pancreatic cancer metabolism. De Nicola. et al. (36) found that oncogenes can activate the antioxidant program of Nrf2 and lead to the occurrence of pancreatic cancer. This article is the most cited in the study of pancreatic cancer metabolism in the United States (cited 1,382 times). Ben et al. (37) found that diabetes is associated with pancreatic cancer risk through a cohort study, which is the most cited article in China and the most cited article in FUDAN UNIV. (cited 411 times).

The top 10 most productive authors all have more than 30 articles in total. Among them, LI (38), the author with the most publications, stated in his magnum opus that the high expression of 5-HT and its receptor HTR2B would increase tumor glycolysis and promote the growth of pancreatic cancer (cited 140 times).

Among the top 10 published institutions, seven are from the United States, while the rest are in China. FUDAN UNIV of China and UNIV TEXAS MD ANDERSON CANC CTR of the United States are the leading institutions of pancreatic cancer metabolism research in the two countries, respectively. UNIV TEXAS MD ANDERSON CANC CTR’s most-cited article reveals oncogenic KRAS-mediated reprogramming of glutamine metabolism in pancreatic cancer (34).

CANCERS, PLOS ONE, and CANCER RESEARCH are the three most published journals. These data will help future scholars choose a journal when submitting manuscripts related to pancreatic cancer metabolism. Amrutkar et al. (39) summarized the gemcitabine metabolism, activation, and chemoresistance entities in pancreatic cancer, and proposed methods to overcome chemoresistance (cited 221 times, most cited in CANCERS). Through systematic review and meta-analysis, Noto et al. (40) believe that taking metformin can reduce the risk of cancer in patients with diabetes, but there is no statistical significance in the study of pancreatic cancer (cited 399 times, most cited in PLOS ONE). Kamphorst et al. (41) found that the scavenging of extracellular proteins is an important mode of nutrient uptake in pancreatic cancer, which can promote glutamine metabolism by synthesizing essential amino acids (cited 433 times, most cited in CANCER RESEARCH).

From the results of co-cited references, the current research on pancreatic cancer metabolism mainly focuses on “nutriment-deficient environment”, “cancer chemoprevention” and “targeting cancer stem cell”. Pancreatic cancer cells can proliferate and metastasize under conditions of nutrient deprivation and hypoxia caused by uncontrolled growth, vascular disturbance, and desmoplastic reactions, mainly relying on the metabolic reprogramming of pancreatic cancer cells (42). Slotwinski et al. (43, 44) review adaptive metabolism and potential therapeutic targets of pancreatic cancer in nutrient-deficient settings. Chemoprevention is the use of natural, synthetic, or biological agents to inhibit tumor progression, which means low risk and no side effects (45). The chemoprevention of pancreatic cancer mainly includes lifestyle, diet, body disorder, dietary compounds, and some chemopreventive agents (such as non-steroidal anti-inflammatory drugs, cyclooxygenase inhibitors, ibuprofen, etc.) (46). Cancer stem cells are considered not only tumor-initiating cells but also cells that promote tumor development and treatment resistance, leading to tumor progression and recurrence (47). At present, the drugs targeting pancreatic cancer stem cells mainly include Reactive oxygen species (ROS) inhibitors, acetylsalicylic acid, mTOR inhibitors, metformin, etc (48).

The results of studying the time when the keywords first appeared and the time flow process of the keywords show that the current hotspots in pancreatic cancer metabolism research are “immunotherapy”, “ferroptosis” and “targeted therapy”. Ferroptosis is a new type of programmed cell death that is iron-dependent and different from apoptosis, necrosis, and autophagy (49). Pancreatic cancer can be linked to ferroptosis through iron metabolism, autophagy, amino acids metabolism, lipids metabolism, and the tumor microenvironment, and can induce ferroptosis in pancreatic cancer cells by targeting these pathways to play a therapeutic role (50). Targeted therapy and immunotherapy have brought hope to cancer patients as emerging treatment options. However, pancreatic cancer, as an extremely malignant tumor with a poor prognosis, has low immunogenicity, and common immune checkpoint blockers such as CTLA-4 or PD-1 inhibitors are ineffective for pancreatic cancer patients (51). In addition, because pancreatic cancer tumor cells carry unique gene drive mutations, there is currently no suitable molecular targeted drug for pancreatic cancer. Recently, scholars have successively discovered MEK1/2 inhibition (MEKi) (52), DNA methyltransferase 1 (DNMT1) (53), and cytoskeleton-associated protein 4 (CKAP4) (54) and other therapeutic targets for pancreatic cancer, but it still needs further clinical verification. Given the unclear treatment plan for pancreatic cancer, some scholars have proposed that targeted metabolism can change the tumor microenvironment and increase immune infiltration through targeted metabolism, transforming pancreatic cancer from drug-resistant tumors to susceptible tumors, and possibly improving the efficacy of immunotherapy (55). The role of traditional Chinese medicine (TCM) in the treatment of tumors cannot be underestimated. In our visualization, curcumin and resveratrol can act through pancreatic cancer metabolic pathways. The effect of curcumin on pancreatic cancer has been studied in great detail. Curcumin can inhibit the proliferation of pancreatic cancer cells through NF-κB pathway (56), DNA damage-mediated G2/M cell cycle arrest (57), PI3K/Akt pathway (58), and other pathways. Furthermore, Masashi Kanai et al. (59) demonstrated through phase I/II clinical trials that curcumin in combination with gemcitabine is feasible and safe for pancreatic cancer. Resveratrol can exert anti-pancreatic cancer effects by inhibiting NFκB activation (60), HIF-1α-induced hypoxia pathways (61), and reducing RYR2 (62) and VEGF-B (63) expression. Although many scholars have proved that some TCM can treat pancreatic cancer through experiments (64), few scholars have studied the mechanism of action of heavy TCM through the metabolic pathway of pancreatic cancer. This suggests that we can take the metabolism of pancreatic cancer as an entry point to provide a more molecular biological basis for the treatment of pancreatic cancer with TCM, and also provide new ideas for the treatment of pancreatic cancer.

For the first time, we systematically visualized and analyzed the articles and trends of pancreatic cancer metabolism in an intuitive way, which can provide ideas for scholars in this field to find research directions in this field. At the same time, our study also has limitations. For example, the articles we study are all based on the WoSCC database, and do not combine multiple databases at the same time; in addition, the literature analysis software CiteSpace and VOSviewer and Bibliometrix Package we use may make the statistical results slightly different due to version problems; Finally, some recently published high-quality articles may not receive the attention we deserve due to their low citation counts.

## Conclusion

As a malignant tumor with an extremely poor prognosis, pancreatic cancer has always been a concern by scholars and clinicians for its diagnosis, treatment, and prognosis. Our bibliometric analysis shows that the number of publications on pancreatic cancer metabolism research is increasing year by year. The USA is the most published country, Li J is the most productive author, FUDAN UNIV is the most published institution and CANCERS is the most published journal. “Immunotherapy”, “ferroptosis” and “targeted therapy” are current research hotspots in pancreatic cancer metabolism. Pancreatic cancer metabolism will provide a new direction for the treatment of pancreatic cancer in TCM and western medicine treatment.

## Data availability statement

The original contributions presented in the study are included in the article/**Supplementary Material**. Further inquiries can be directed to the corresponding author.

## Author contributions

All authors contributed to the article and approved the submitted version. QS and CZ designed the thesis idea and wrote the original draft. XJ and FL proposed the methodology. JL, XZ, and GE prepared the figures. BP reviewed and edited the manuscript.

## Funding

Supported by the Scientific and Technological Innovation Project of China Academy of Chinese Medical Sciences (Major research project of Oncology, No. CI2021A01805). Beijing Municipal Science and Technology Commission capital Clinical Diagnosis and treatment technology research and demonstration application special project (General Program, No. Z191100006619022).

## Conflict of interest

The authors declare that the research was conducted in the absence of any commercial or financial relationships that could be construed as a potential conflict of interest.

## Publisher’s note

All claims expressed in this article are solely those of the authors and do not necessarily represent those of their affiliated organizations, or those of the publisher, the editors and the reviewers. Any product that may be evaluated in this article, or claim that may be made by its manufacturer, is not guaranteed or endorsed by the publisher.
